# Daily Rhythms of Blood Parameters in Broiler Chickens Reared under Tropical Climate Conditions

**DOI:** 10.5334/jcr.151

**Published:** 2017-08-10

**Authors:** Harold Kuta Makeri, Joseph Olusegun Ayo, Tagang Aluwong, Ndazo Salka Minka

**Affiliations:** 1 Department of Animal Health and Husbandry, College of Agriculture and Animal Science, Division of Agricultural Colleges, Ahmadu Bello University, Mando-Kaduna, NG; 2 Department of Physiology, Faculty of Veterinary Medicine, Ahmadu Bello University, Zaria, NG

**Keywords:** blood, broiler chickens, circadian rhythm, natural photoperiod, tropical climate

## Abstract

Several studies carried out on humans and other mammals show that the temporal organisation of haematological parameters in the blood exhibit daily rhythms; however, such studies have been rare in poultry reared under a natural photoperiod. The present study investigated the occurrence of daily rhythms in blood parameters of broiler chickens kept under tropical climatic conditions. Ten 6–7-week-old broiler chickens served as subjects of the study. They were kept in standard individual cages under natural light-dark cycle and given access to feed and water *ad libitum*. Two milliliters of blood was collected from each bird via intravenous cannulae inserted into the wing vein. The blood samples were collected every 4 h over a 24-h period, starting from 09:00 h on the first day and completed at 09:00 h on the second day. The blood samples were analysed for erythrocyte, total and differential leucocyte counts. A trigonometric statistical model according to the single cosinor procedure was used to describe the periodic parameters and their acrophases, and ANOVA was used to determine significant differences. The results demonstrated the existence of daily rhythms in packed cell volume, haemoglobin, white blood cell, red blood cell, heterophil, lymphocyte, eosinophil and monocyte counts, while total protein displayed no rhythm. The characteristics of the haematological parameters showed that the acrophases were restricted to the light phase of the light/dark cycle, precisely at 09:00 h, except for eosinophil and heterophil counts, which had acrophases at 21:00 h. The amplitudes of the blood parameters varied, with packed cell volume having the greatest amplitude of 4.2 ± 0.5, closely followed by lymphocyte (3.4 ± 0.5) and heterophil (2.3 ± 0.2) counts. In conclusion, the results of the study demonstrated the existence of daily rhythms with diurnal acrophases in blood parameters of broiler chickens kept under natural photoperiods and tropical conditions.

## Introduction

Circadian rhythms, which are about-24-hour oscillations in behaviour and physiology, are reflected in all cells of the body, aimed at optimising cellular functions and meeting environmental challenges associated with the solar day [1]. The multi-oscillatory network in all levels of cell line is coordinated by the master pacemaker clock located in the suprachiasmatic nucleus (SCN) of the hypothalamus, which directs an animal’s rhythmic expression of physiological functions and behaviour via a hierarchical system [1,2,3].

The circadian system has been extensively studied in mammals [4,5]; however, in poultry such studies are limited [6,7]. The avian circadian system is more complex than that of mammals. It is composed of circadian oscillators that reside within the pineal gland, retina and SCN. The SCN in birds consist of two structures, the visual (v-SCN) and the medial (m-SCN) SCN [6]. Birds have extra-ocular photoreceptors; and, correspondingly, they have independently-regulated pacemakers, which are lacking in mammals [6]. Thus, the study of chronophysiology in avian species may require more attention.

Some investigations have been carried out on the temporal organisation of haematological parameters in humans [8] and livestock [7,9,10,11]. Results of the studies show that the daily rhythms of circulating blood cells are complex in nature, and that the number of circulating blood cells in the peripheral blood shows daily rhythms in the majority of cell lines.

The clinical significance of haematological studies in poultry production has been increasing, particularly in those concerned with the determination of normal values of blood parameters and investigation under several disease and environmental conditions [12]. Although extensive studies on peripheral rhythms of many physiological processes have been reported in mammals, such studies in poultry are limited [7]. In addition, the majority of daily rhythms studied were conducted under thermoneutral or laboratory-regulated environments. There is a growing body of evidence that rhythm patterns often differ between thermoneutral or laboratory-controlled environments and natural conditions [7,13,14,15]. Besides, studies on daily rhythms during natural photoperiod under tropical conditions are generally limited [7,15]. The present study investigated the existence of daily rhythms in blood parameters and their characteristics in broiler chickens reared under natural tropical conditions.

## Materials and Methods

### Study area and management of birds

The experiment was conducted at the experimental poultry pen of the Livestock Farm of College of Agriculture and Animal Science, Mando-Kaduna (11°10/N, 07°38/E), located in the Northern Guinea Savannah zone of Nigeria.

Ten, 6–7-week-old, clinically healthy, male broiler chickens with mean live body weights of 2.0 kg (1.80 – 2.25 kg) served as subjects of the study. The birds were kept and managed individually in cages of 42 × 35 × 35 cm dimensions under natural conditions of 12:30/11:30 light:dark circle. The health status of the birds was assessed twice weekly by evaluation of body temperature and observation of innate and feeding behaviours. The broiler chickens were fed *ad libitum* on an isocaloric and iso-nitrogenous diet of 2800 kCal/kg and crude protein (CP) 21%, respectively, and given access to water *ad libitum*. The handling and other experimental procedures of the birds were humanely carried out according to International Guideline for the Care and Use of Laboratory Animals (2011). The research was approved by the Research and Postgraduate Ethical Committee on Animal Welfare, Department of Physiology, Faculty of Veterinary Medicine, Ahmadu Bello University, Zaria, Nigeria (Ethical No. P15VTPY9001 – 2016). Mortality was not observed among the broiler chickens that served as subjects during the study period.

### Measurements of thermal data

Thermo-hygrometric readings were taken at three-hourly intervals to correspond with blood sampling times for 24 hours, with the aid of a dry- and wet-bulb thermometer (DTH 1; Clarke International Ltd., Essex, UK), placed in the middle of the pen, 1.5 m high from the floor.

### Collection of blood samples

Brachial veins of the birds were cannulated with 24 G × 0.7 × 19 mm (Lars Medicare Pvt. Ltd., India) vein cannulas and stabilised using subcutaneous stay sutures (catgut). About 2 mL of blood samples was drawn at three-hour intervals over a 24-h period (starting from 09:00 h on day 1 and finishing at 09:00 h on day 2) into tubes; one, containing ethylene diamine tetra-acetic acid (EDTA) at the ratio of blood to anticoagulant of 1–2 mg/ml and the other without EDTA. The blood samples containing EDTA were used to determine red blood cell (RBC) count, packed cell volume (PCV), haemoglobin (Hb) concentration, and white blood cell (WBC) and differential counts [16]. Blood samples were analyzed within 1–2 hours of collection, for PCV, Hb concentration, and RBC count using an automated cell counter (Celltac MEK-6108 K; Nihon-Kohdon, Tokyo, Japan). The differential leucocyte counts were performed using the straight-edge method described by Schalm *et al*. [16]. Plasma obtained from centrifuged blood without EDTA was used for TP analysis a day after collection of the blood. The TP was analysed using a refractometer.

### Statistical analysis

The results obtained were expressed as mean ± SEM. Values of *P* less than 0.05 were considered statistically significant. One-way repeated-measures analysis of variance (ANOVA) was used to determine a statistical significant effect of time on the studied parameters. Data were analysed using the software STATISTICA 5.5 (StatSoft Inc., USA). A trigonometric statistical model to the average values of each time series was applied, and the periodic phenomenon was described analytically by individuating the main rhythmic parameters according to the single cosinor procedure [11,17]. Three rhythmic parameters were determined: mean level (mesor), amplitude and acrophase (time at which the peak of rhythm occurred). The difference between times of measurement was tested by Student’s paired *t*-test. Values of P less than 0.05 were considered significant.

## Results

### Mean, maximum and minimum values of blood parameters of broiler chickens housed under a natural photoperiod and thermal environmental data inside the pen

Table 1 shows the mean, maximum and minimum values of blood parameters of broiler chickens, and the thermal environmental data inside the pen. All the blood parameters measured displayed individual variation between the birds. The ambient temperature (AT), relative humidity (RH) and temperature-humidity index (THI) had mean values of 24.5 ± 0.6°C, 95.40 ± 10.5% and 76.90 ± 7.4, respectively.

**Table 1:** Mean, maximum and minimum haematological parameters in broiler chickens and thermal environmental data of the pen under natural light-dark cycle.

Parameters	Mean ± SEM	Maximum	Minimum

PCV, %	25.92 ± 1.5	30.20	21.90
Hb, g/dL	8.63 ± 0.8	10.03	7.26
TP, g/dL	3.51 ± 0.5	3.72	3.44
WBC, × 10^9^/L	13.78 ± 1.8	14.78	11.18
RBC, × 10^12^/L	4.27 ± 0.3	5.14	3.74
LYMP, %	85.40 ± 7.5	89.00	82.3
HET, %	12.20 ± 2.2	14.60	10.10
EOS, %	0.86 ± 0.3	1.75	0.31
MON, %	1.50 ± 0.03	1.90	0.60
H/L ratio	0.14 ± 0.02	0.18	0.11
AT, °C	24.5 ± 0.6	29.01	20.10
RH, %	95.40 ± 10.5	100.00	85.00
THI	76.90 ± 7.4	81.64	70.12

PCV = Packed cell volume, Hb = Haemoglobin, TP = Total protein, WBC = White blood cell, RBC = Red blood cell, LYMP = Lymphocyte, HET = Heterophil, EOS = Eosinophil, MON = Monocyte, H/L = Heterophil/lymphocyte ratio, AT = Ambient temperature, RH = Relative humidity, THI = Temperature humidity index.

### Daily rhythms of packed cell volume, haemoglobin and total protein

**Figure 1:**
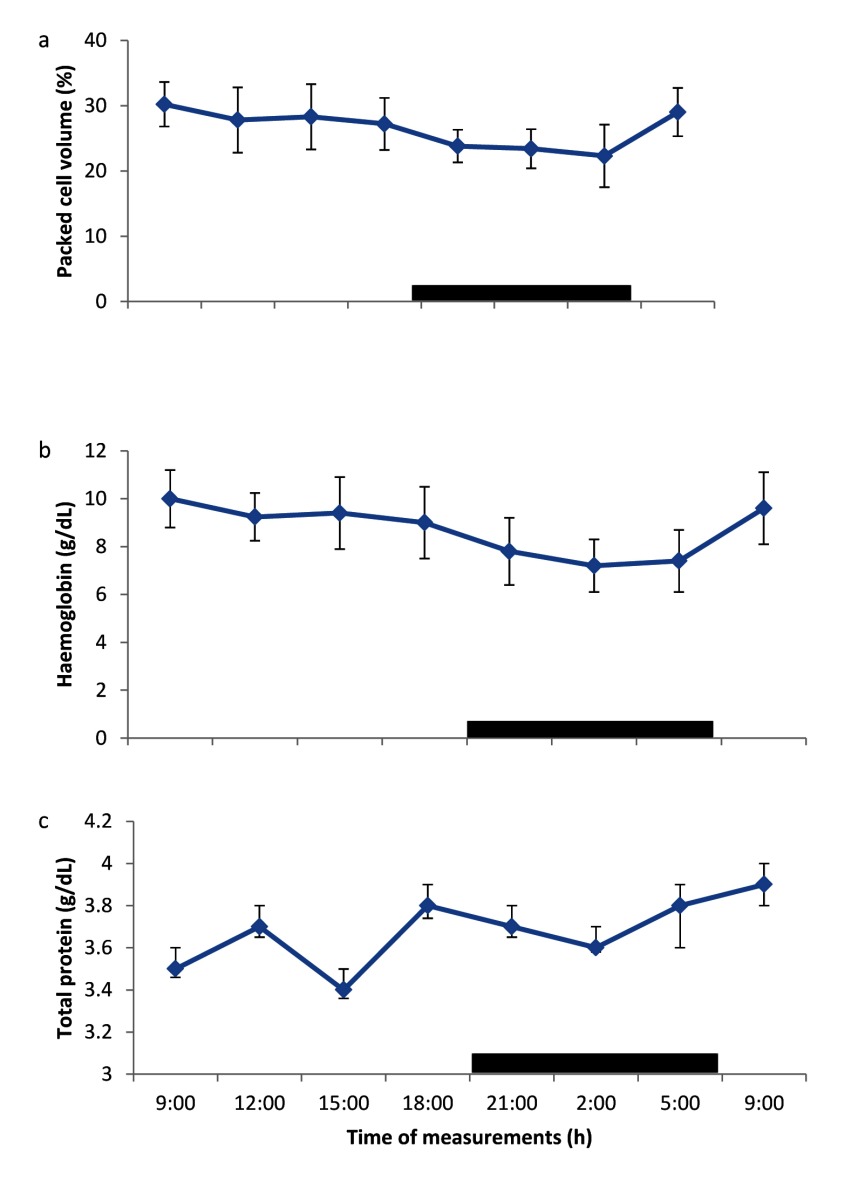
Daily rhythm of **a)** packed cell volume, **b)** haemoglobin and **c)** total protein in broilers kept under natural photoperiod. Notes: Each data point represents the mean ± SEM of 10 birds at each period of measurements. The black horizontal bars denote the dark phases of the prevailing light-dark cycle. Measurements were made twice on separate dates at 3-h intervals for a period of 24 hours.

The PCV exhibited daily rhythms with an ascent during the photophase (09.00 – 18.00 h) and a descent during the scotophase (dark phase) (21.00 – 05.00 h) (Figure 1a). The Hb concentration exhibited weak daily rhythms (Figure 1b), with an ascent and descent phases similar to those of PCV. The daily rhythms of TP concentration was not clearly defined; thus, did not show any rhythm, rather the TP concentration fluctuated between the maximum and minimum values during both the light and dark cycles (Figure 1c).

### Daily rhythms of white and red blood cell counts

**Figure 2:**
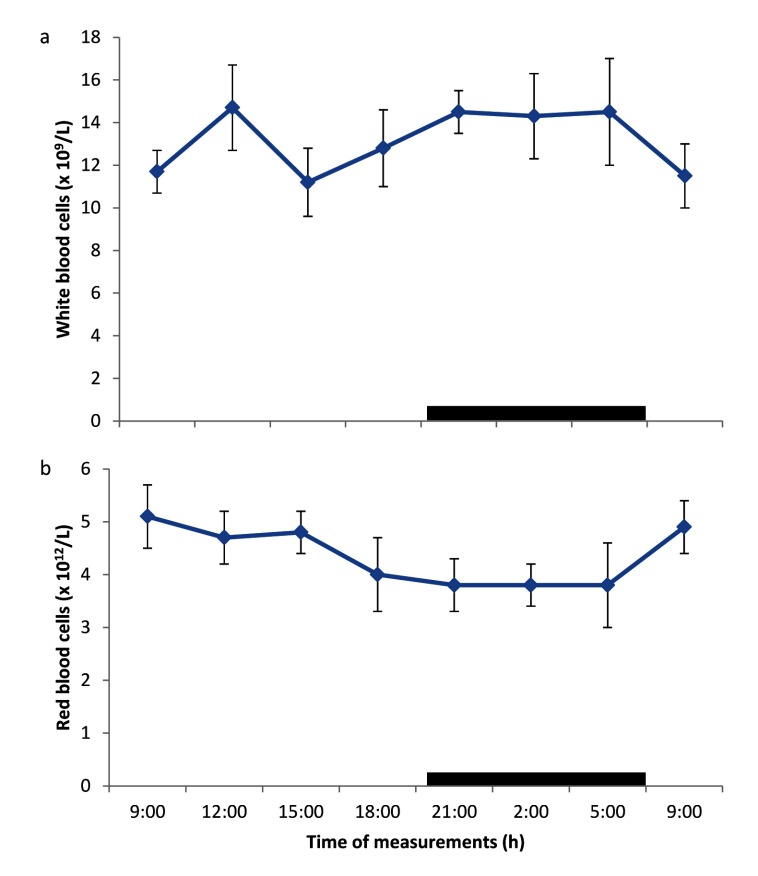
Daily rhythm of **a)** white blood cell and **b)** red blood cell counts in broilers kept under natural photoperiod. Notes: Each data point represents the mean ± SEM of 10 birds at each period of measurements. The black horizontal bars denote the dark phases of the prevailing light-dark cycle. Measurements were made twice on separate dates at 3-h intervals for a period of 24 hours.

The WBC counts showed the existence of daily rhythms (Figure 2a), with a progressive ascent during the scotophase (18.00 – 21.00 h), while daily rhythmicity of RBC was weak but defined, with an ascent during the photophase (09.00 – 15.00 h) and a descent during the scotophase (18.00 – 21.00 h) (Figure 2b).

### Daily rhythms of leucocyte counts

**Figure 3:**
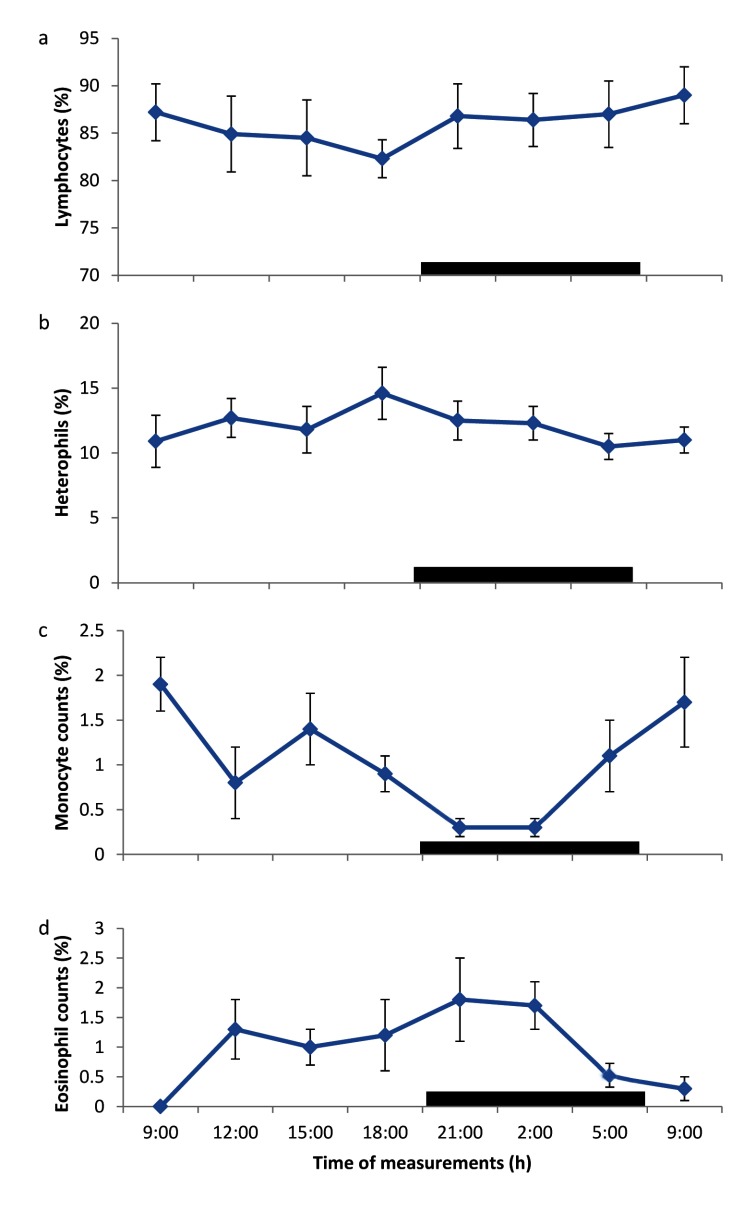
Daily rhythm of differential leucocyte counts in broiler chickens kept under natural photoperiod. Notes: Each data point represents the mean ± SEM of 10 birds at each period of measurements. The black horizontal bars denote the dark phases of the prevailing light-dark cycle. Measurements were made twice on separate dates at 3-h intervals for a period of 24 hours.

The daily rhythms of differential leucocyte counts are shown in Figures 3a–3d. The lymphocyte counts recorded in broiler chickens over the 24-h study period demonstrated a clear daily rhythm; with an ascent during the photophase, and a descent during the scotophase (Figure 3a). The heterophil counts also showed a clear daily rhythm, but with an ascent during the scotophase and a descent during the photophase (Figure 3b). The daily rhythms observed in eosinophil counts exhibited an ascent during both light and dark phases (12.00 – 21.00 h) (Figure 3c), while the monocyte counts showed daily rhythm with higher values during the photophase and lower values during the scotophase (Figure 3d).

### Characteristics of haematological parameters

The mesor, amplitude and acrophases of the haematological parameters recorded in the broiler chickens during the study are shown in Table 2. The majority of the blood samples had acrophases at 09:00 h, while the amplitude of the blood parameters differed in greatness, with greater amplitude recorded in PCV (Table 2).

**Table 2:** Characteristics of haematological parameters in broiler chickens kept under natural light/dark cycle. Measurements were made at 3-h intervals for a period of 24 hours.

Blood parameters	Mesor	Amplitude	Acrophase (h)

PCV, %	25.2 ±1.3	4.2 ± 0.5	09:00 ± 0.13
Hb, g/dL	8.2 ± 0.7	1.4 ± 0.4	09:00 ± 0.16
WBC, × 10^9^/L	13.47 ± 2.2	1.8 ± 0.5	09:00 ± 0.18
RBC, × 10^12^/L	4.18 ± 1.5	0.7 ± 0.4	09:00 ± 0.25
HET, %	11.6 ± 1.0	2.3 ± 0.2	18:00 ± 0.23
LYMP, %	86 ± 12.5	3.4 ± 0.5	09:00 ± 0.17
MONO, %	0.99 ± 0.2	0.7 ± 0.01	09:00 ± 0.21
EOS, %	0.82 ± 0.3	0.7 ± 0.02	21:00 ± 0.30
H/L ratio	0.12 ± 0.03	0.04 ± 0.01	18:00 ± 0.20

PCV = Packed cell volume, Hb = Haemoglobin, TP = Total protein, WBC = White blood cell, RBC = Red blood cell, LYMP = Lymphocyte, HET = Heterophil, EOS = Eosinophil, MON = Monocyte, H/L = Heterophil/lymphocyte ratio, AT = Ambient temperature, RH = Relative humidity, THI = Temperature humidity index.

## Discussion

One of the most frequently analysed samples in clinical and laboratory investigations is blood sample, especially the PCV, but there is paucity of information on rhythmicity of blood parameters. From the results obtained in broiler chickens in the present study, the PCV, Hb and RBC showed the existence of daily rhythms; with peak (acrophase) during the light phase (09:00 h) and descent during the dark phase, which was similar to the rhythm of other blood parameters reported for the eagle [18]. The PCV, Hb and RBC rhythms obtained in broiler chickens in the present study were similar to those obtained by other investigators, who showed the existence of daily rhythms of PCV, Hb and RBC in humans [19,20,21], cows [22], rodents [23] and horses [13], with peak values occurring between 09:00 – 12:00 h. However, the PCV result of the present study disagrees with the findings of Giannetto and Piccione [24], who demonstrated the absence of rhythm in PCV values in *Bos taurus*. Although Durotoye *et al*. [25] reported some pattern of diurnal variation of PCV, Hb and RBC values in local chickens, their data were not fitted into cosinor analysis for rhythm study. Besides, the study was carried out mainly on indigenous chickens in the Southern Guinea Savannah zone of Nigeria. The finding that PCV, Hb and RBC peaked during the light and dark hours in both diurnal and nocturnal mammals [13,19,20,21,22,23,26], and presently in poultry, suggests that the PCV, Hb and RBC were not modulated by physical activity. The PCV, Hb and RBC results obtained in the present study showed, for the first time, that the blood parameters of broiler chickens reared under a natural light-dark cycle in the Northern Guinea Savannah zone of Nigeria exhibited daily rhythmicity. The lack of information in the available literature on daily rhythm of Hb concentration in broiler chickens did not warrant any comparison with values, obtained in the present study, thus further investigation is required.

The mesor of PCV and Hb values of 25.9 ± 1.3% and 8.2 ± 0.7 g/dL, respectively, obtained in broiler chickens in the present study, were lower than the overall daily ranges of PCV and Hb values, recorded previously in indigenous chickens in Nigeria [25] under tropical conditions; and they also exhibited individual and diurnal variations. However, the Hb value obtained in the present study was similar to that of 8.9–9.8 g/dL, reported in broiler chickens reared under tropical conditions [27]. The RBC counts fluctuated between 3.73 × 10^12^/L – 5.14 × 10^12^/L; with a mesor of 4.18 ± 1.5 × 10^12^/L, which was higher than the range values of 2.18 × 10^12^/L – 2.48 × 10^12^/L, recorded in indigenous chickens, reared under tropical conditions [23,28]; and the values of 2 – 3 × 10^12^/L, recorded in broilers in Nigeria [27,28]. The difference in the mean and mesor, obtained in the present study may be because the data from the previous study were not fitted into cosinor analyses; thus, limiting the clinical application of such base-line data.

The mean TP value of 3.48 ± 0.3 g/dL recorded in broilers in the present study was similar to that of 2.9 – 3.5 g/dL, reported for broiler chickens [29], but lower than the value of 6.18 mg/dL obtained in chickens under tropical conditions [25]. The result showed that TP values in broilers did not exhibit a clear rhythm, rather they demonstrated both ascent and descent phases during light and dark cycle, and did not exhibit significant variations during the study period. The result of the present study was similar to that of Durotoye *et al*. [25], who observed insignificant variation in TP values of indigenous chickens in Nigeria. Similarly, the lack of daily rhythm of TP was reported in *Bos taurus* [24]. The absence of daily rhythm of TP concentration in broiler chickens demonstrated that the rhythm was not influenced by light:dark cycle or activity, and probably by any endogenous factor. Thus, further studies are required in this direction.

The daily rhythm in WBC counts was the composite of the daily rhythm of different types of leucocytes, some of which have different circadian phasing [21]. The mesor of WBC count of 13.47 ± 2.2 × 10^9^/L, recorded in broiler chickens in the present study was significantly higher than the count of 2.2 – 7.5 × 10^9^/L obtained in broilers [29]; but lower than the mean WBC count of 24.6 × 10^9^/L, recorded by Durotoye *et al*. [25] in indigenous chickens. The WBC count of broilers in the present study exhibited both individual and diurnal variations, similar to those reported by Durotoye *et al*. [25] in indigenous chickens. The WBC count in broilers exhibited rhythm with an ascent during the dark phase which progressed toward morning (21.00 h – 12.00 h) and a descent phase from 15.00 – 18.00 h, which was similar to the peak periods (21:00 h to 05: 00 h), observed in WBC counts in humans [21]. The pattern of diurnal fluctuation of WBC count in broilers in the present study was contrary to the finding of Piccione *et al*. [13], who reported no daily rhythm in the WBC count of horses. Although the WBC rhythm in broiler chickens was similar to the WBC daily rhythm displayed in humans [19], owl monkeys [30] and rodents [23], the acrophase of WBC in rodents was observed during the light phase, apparently due to their nocturnal lifestyle.

The lymphocyte counts obtained in broiler chickens in the present study, which fluctuated between 78 – 90 % and with a mesor of 86 ± 12.5%, were higher than the range values of 50 – 62%, recorded in broiler chickens in Nigeria [27,29]. The lymphocyte counts, like the heterophil counts, showed the existence of a clear daily rhythm. However, the lymphocyte counts displayed a descent phase during the afternoon period (12.00 – 18.00 h), and an ascent during the dark phase (21.00 h) up to the morning hours. The result demonstrated an inverse relationship with those of the heterophil count. The daily rhythm of lymphocyte count in the present study was similar to the finding of Cahyaningsih *et al*. [31], who demonstrated that daily rhythm of lymphocyte counts in chickens, kept under ambient temperature of 23°C displayed an ascent phase at night (03:00 h) and early morning (11:00 h). Similarly, the daily rhythm of lymphocyte counts in humans was shown to peak at mid-night (24:00 h) [23], while in cows the rhythm was observed to peak by late afternoon (16:00 h) [24].

In general, the daily rhythm of lymphocyte counts in diurnal mammals and poultry has acrophases during the dark phase; while in nocturnal animals (mice), they display peak values during the light phase [32]. The present result confirmed the finding that immunity of chickens has some diurnal rhythms, which have been reported in mammals [31]. Although previous studies were conducted under thermoneutral conditions, the present result showed that, even under natural photoperiod, the immunity of broiler chickens exhibited some rhythms, with the acrophsae displaying a biphasic pattern as earlier reported in chickens [31]. This finding may be of clinical significance, when administering vaccines and other related therapeutic and prophylactic agents to birds.

Heterophils are commonly involved in fighting infection and healing process and they function more effectively in combination with lymphocytes. The heterophil counts in the present study fluctuated between 10 – 14.6%; with a mean value of 12.2 ± 2.2%, and mesor of 11.6 ± 1.0%. The mean base-line value was similar to the value of 11% reported in broilers [29]. The heterophil counts in broiler chickens exhibited a clear daily rhythm. The pattern of rhythms showed a gradual increase in heterophil counts from 12.00 h to a peak at 18.00 h, and progressively decreased from 21.00 h – 09.00 h. The rhythm of heterophil counts in broilers was similar to that of neutrophil counts reported in humans, where the peak values were recorded late afternoon (19:00 h) [21]. However, in cows the neutrophil counts had a peak during the dark phase (05:00 h) [22]. The result of heterophil counts in the present study, for the first time, demonstrated the existence of daily rhythms in broiler chickens, reared under natural photoperiod in the Northern Guinea Savannah zone. The lack of information in the available literature on daily rhythms of heterophil counts in poultry did not warrant any comparison with our result. Thus, further studies are required in this direction.

The eosinophil counts of 0 – 1.75% recorded in the present study, which had a mesor value of 0.86 ± 0.3%, were similar to the eosinophil counts of 0 – 2 %, reported by Nanbol *et al*. [28]; but lower than the counts of 3 – 5% [27,29], reported in other broiler chickens in Nigeria. The eosinophil counts displayed rhythmicity with higher values during the dark phase and lower values during the morning hour; which was similar to rhythms of eosinophil counts, reported in cows [22] and Owl monkeys [30]. The lack of information on daily rhythm of eosinophil counts in poultry did not allow any comparison with the result of the present study.

The mean monocyte counts recorded in broilers in the present study were similar to the values of 2 – 5%, reported in broiler chickens in Nigeria [28]; however, the mesor of 0.99 ± 0.2% recorded in the broiler chickens in the present study was lower than the mean values. The monocyte counts also exhibited individual variability, similar to the result reported by Durotoye *et al*. [25] and Nanbol *et al*. [28] in local chickens in Nigeria. The daily rhythm observed in monocyte counts suggested an increase in the counts during the photophase and a decrease during the dark phase.

The fact that higher haematological data were not recorded at the time that the ambient temperature was at its peak in the afternoon suggested that the daily rhythms of the haematological parameters in broilers were not controlled by the ambient temperature.

The result of the amplitude of daily rhythm of haematological values in the broilers showed that each blood parameter had a different amplitude, which ranged from 0.24 – 4.2. However, the RBC, monocyte and eosinophil counts had similar amplitudes of 0.7 – 0.73. Similar, variations in amplitude of blood parameters has been reported in different livestock [7,22,24]. Of the parameters measured, the PCV and lymphocyte counts had the greatest amplitude, while the lowest amplitude was recorded in TP, apparently because the TP did not exhibit any rhythm or variability. It has been shown that greater amplitude is accompanied by a reduction in mesor [4]. In the present study, haematological parameters with larger values tended to have greater amplitude, while those with smaller values had the tendency to have lower amplitudes. In general, greater amplitude was observed to be a consequence of wide range of ambient temperature [7,11,15]. In the present study, the ambient temperature exhibited less variation, which, apparently, resulted in lower amplitude values of the blood parameters measured.

## Conclusion

The results demonstrated the existence of daily rhythms in blood parameters of broiler birds kept under natural photoperiods. The majority of the blood parameters measured had acrophases restricted to the light phase of the light-dark cycle. The results may be beneficial in re-evaluating base-line data of haematological parameters, improvement performance, and clinical diagnosis, treatment and prevention of diseases of broiler chickens. The knowledge from cellular rhythmicity may have broader biological implications, including reproductive biology.

## References

[1] Matsui MS, Pelle E, Dong K, Pernode N (2016). Biological Rhythms in the Skin. Int J Mol Sci.

[2] Refinetti R (2010). The circadian rhythm of body temperature. Front Biosci.

[3] Giannetto C, Carcangiu V, Luridiana S, Fazio F, Mura MC, Parmeggiani A, Piccione G (2016). Causal link of total locomotor activity, melatonin and rectal temperature daily rhythm in small ruminants. J Appl Biomed.

[4] Piccione G, Refinetti R (2003). Thermal chronobiology of domestic animals. Front Biosci.

[5] Refinetti R (2016). Circadian Physiology.

[6] Bell-Pedersen D, Cassone VM, Earnest DJ, Golden SS, Hardin PE, Thomas TL, Zoran MJ (2005). Circadian rhythms from multiple oscillators: lessons from diverse organisms. Nat Rev Genet.

[7] Minka NS, Ayo JO (2016a). Daily rhythms of colonic temperature and circulating blood enzymes, urea and calcium in Japanese quail (Coturnix coturnix japonica) under natural cold-dry (harmattan) and hot-dry conditions. Bio Rhythm Res.

[8] Berger J (1983). Seasonal influences on circadian rhythms in the blood picture of SPF rats housed under artificial illumination. Folia Haematol.

[9] Piccione G, Caola G (2002). Biological rhythm in livestock. J Vet Sci.

[10] Piccione G, Caola G, Mortola JP (2005a). Scaling the daily oscillations of breathing frequency and skin temperature in mammals. Comp Biochem Physiol. Part A.

[11] Piccione G, Gianesella M, Morgante M, Refinetti R (2013). Daily rhythmicity of core and surface temperatures of sheep kept under thermoneutrality or in the cold. Res Vet Sci.

[12] Surai PF (2016). Antioxidant systems in poultry biology: superoxide dismutase. J Anim Res and Nutri.

[13] Piccione G, Fazio F, Giudice E, Grasso F, Morgante M (2005b). Nycthemeral change of some haematological parameters in horses. J Appl Biomed.

[14] Pita R, Mira A, Beja P (2011). Circadian activity rhythms in relation to season, sex and interspecific interactions in two Mediterranean voles. Anim Behav.

[15] Minka NS, Ayo JO (2016b). Effects of cold-dry (harmattan) and hot-dry seasons on daily rhythms of rectal and body surface temperatures in sheep and goats in a natural tropical environment. J circadian rhythms.

[16] Schalm OW, Jain NC, Carroll EJ (1975). Veterinary haematology.

[17] Refinetti R, Cornélissen G, Halberg F (2007). Procedures for numerical analysis of circadian rhythms. Biol Rhythm Res.

[18] Garcia-Rodriguez T, Ferrer M, Recio F, Castroviejo J (1987). Circadian rhythms of determined blood chemistry values in Buzzards and Eagle owls. Comp Biochem Physiol.

[19] Pocock SJ, Ashby D, Shaper AG, Walker M, Broughton PMG (1989). Diurnal variations in serum biochemical and haematological measurements. J Clin Pathol.

[20] Haus E, Touitou Y, Haus E (1992). Chronobiology of circulating blood cells and platelets. Biological Rhythms in Clinical and Laboratory Medicine.

[21] Redfern PH, Lemmer B (2013). Physiology and Pharmacology of Biological Rhythms.

[22] Giudice A, Crispo A, Galdiero M, D’Arena D, Tecce MF, Grimaldi M, Amore A, Esposito E, Montella M (2014). Metabolic syndrome, insulin resistance, circadian disruption, antioxidants and pancreatic carcinoma: an overview. J Gastroenterol Liver Dis.

[23] Sanni AA, Oyedokun OR, Alaka OO (2000). Preliminary observations on diurnal rhythm in the haematological parameters of male African giant rats (*Cricetomys gambianus*, Waterhouse). Afri J Biomed Res.

[24] Giannetto C, Piccione G (2009). Daily rhythms of 25 physiological variables in Bos taurus maintained under natural conditions. J Appl Biomed.

[25] Durotoye LA, Fadairo MO, Avwemorue AK (2000). Diurnal variation in blood parameters in the chicken in the hot tropical climate. Afri J Biomed Res.

[26] O’Neill JS, Reddy AB (2011). Circadian clocks in human red blood cells. Nature.

[27] Onibi GE, Bobadoye AO, Folorunso OR (2011). Haematological indices, serum cholesterol and meat quality of broiler chickens fed diets with palm oil sludge substituting maize. Agric Biol J North America.

[28] Nanbol DL, Duru BN, Nanbol HD, Abiliu CA, Anueyegu DM, Kumbish PR, Solomon M (2016). Establishment of reference values for some biochemical and haematological parameters for broilers and layers in Plateau state Nigeria. Vom J Vet Sci.

[29] Adeyemo IA, Sani A (2013). Haematological parametres and serum biochemical indices of broiler chickens fed aspergillus niger hydrolyzed cassava peel meal based diet. Int J Agric Pol Res.

[30] Klein R, Bleiholdes B, Jung A, Erkert H (1985). Diurnal variation of several blood parameters in the owl monkey, *Aotus trivirgatus griseimembra*. Folia Primatol.

[31] Cahyaningsih U, Kondo Y, Abe A, Tanabe A (1990). The diurnal rhythms in lymphocyte counts and antibody formation in Chicks. Jap Poult Sci.

[32] Kawate T, Abo T, Hinuma S, Kumagai K (1981). Studies on the periodicity of the immune response. II. Co-variations of murine T and B cells and a role of corticosteroid. J Immunol.

